# Interstitial microdeletion of the 1p34.3p34.2 region

**DOI:** 10.1002/mgg3.409

**Published:** 2018-05-03

**Authors:** Joseph E. Jacher, Jeffrey W. Innis

**Affiliations:** ^1^ Department of Pediatrics and Communicable Diseases Division of Pediatric Genetics Metabolism & Genomic Medicine University of Michigan Ann Arbor Michigan

**Keywords:** 1p34.2, 1p34.3, chromosome microarray, microdeletions, *SNIP1*

## Abstract

**Background:**

Interstitial microdeletions of chromosome 1p34.3p34.2 are rare, but are continuing to be identified by the use of chromosome microarray. There have been fewer than 10 individuals identified who have deletions of the 1p34.3p34.2 region; all of these previously described individuals have deletions of the *AGO1, AGO3, GRIK3, SLC2A1*, or *RIMS3* genes. Haploinsufficiency of these genes has been associated with neurodevelopmental delays.

**Methods:**

Chromosome microarray, quantitative PCR, and fluorescence in situ hybridization were performed with DNA extracted from peripheral blood.

**Results:**

Chromosome microarray identified a 2.3 Mb 1p34.3p34.2 one copy deletion in our patient with global developmental delay, mild intellectual disability, delayed bone age, bilateral vesicoureteral reflux, vocal cord paralysis, right aberrant subclavian artery, kyphoscoliosis, bilateral metatarsus adductus, and valgus knee deformity. This deletion was confirmed by quantitative PCR and does not include the *AGO1, AGO3, GRIK3, SLC2A1*, or *RIMS3* genes. Subsequent FISH testing of the parents was negative.

**Conclusion:**

Haploinsufficiency of the 1p34.3p34.2 region, including the *SNIP1* gene and excluding the five genes listed above, is responsible for the neurocognitive delays and other symptoms as identified in our patient.

## 1 INTRODUCTION

### 1.1 Background

Interstitial microdeletions of the short arm of chromosome 1 are exceedingly rare, but are continuing to be identified by the use of chromosome microarray. In the 1p34.3p34.2 region of the genome there have been several genes identified to cause neurodevelopmental delays. The *AGO1* (OMIM 606228) and *AGO3* (OMIM 607355) genes when deleted have been hypothesized to be associated with developmental delays, hypotonia, and poor feeding (Tokita et al., 2015). Deletions of the GRIK family genes have been associated with intellectual disability (Takenouchi et al., 2013). The *GRIK3* gene (OMIM 138243) located at 1p34.3 has been reported to be a candidate gene for not only developmental delay and intellectual disability, but also schizophrenia by linkage studies (Takenouchi et al., 2013; Delisi et al., 2002). Haploinsufficiency of the *SLC2A1* gene (OMIM 138140) has been well reported to be a cause for glucose transport type 1 deficiency syndrome 1 (Vermeer et al., 2007). Patients with a deletion of this gene have been described to have severe neurocognitive delays, seizures, microcephaly, and dysmorphic features (Lee, Kim, Kim, & Lee, 2015). The *RIMS3* gene (OMIM 611600) is thought to be a candidate gene for autism spectrum disorder (Kumar et al., 2009).

In this paper, we report a female who has a history of global developmental delay, mild intellectual disability, delayed bone age, bilateral vesicoureteral reflux, vocal cord paralysis, right aberrant subclavian artery, kyphoscoliosis, bilateral metatarsus adductus, and valgus knee deformity. She was identified to have a 2.3 Mb interstitial deletion at 1q34.3q34.2 that does not interrupt the *AGO1, AGO3, GRIK3, SLC2A1*, or *RIMS3* genes, but does affect 43 RefSeq genes including two OMIM disease genes.

### 1.2 Case presentation

The female patient was born without a history of in utero teratogenic exposure to a G3P3 mother and nonconsanguineous parents at 40–3/7 weeks gestation. The birth weight was 6 pounds 2 ounces and birth length was 19 inches. There was no history of hypotonia or other neonatal concerns.

A bone age study was performed at 5‐years‐1‐month of age. According to the hand standards of Greulich and Pyle, the patient's bone age corresponded to a 2‐year‐6‐month‐old female (−2.84 standard deviations [*SD*]). The patient had a history of bilateral vesicoureteral reflux (grade IV on the left). She underwent cystoscopy and bilateral extravesical ureteral reimplantation at the age of 5 years 3 months. At 7 years she was found to have a 2/6 variable musical heart murmur and had a normal EKG. An echocardiogram was not performed and she was given a diagnosis of Still's murmur. At 13 years 8 months of age, she was noted on physical examination to have metatarsus adductus, valgus knee deformity, scoliosis (dextrocurve of 13 degrees from T1 to the thoracolumbar transitional vertebra, levocurvature of seven degrees from there to the lumbosacral transitional vertebra), lumbar lordosis (62°), thoracic kyphosis (53°), and a shallow sacral dimple. Laryngoscopy was performed at 13 years 10 months of age because of a history of choking and revealed left vocal cord paresis, adenotonsillar hypertrophy, and a possible left vallecular cyst. CT of her head, neck, and thorax revealed no acute intracranial abnormalities, although she was noted to have an aberrant right subclavian artery from a left aortic arch. Given the concerns for choking and the adenotonsillar hypertrophy, the patient underwent adenotonsillectomy at 13 years 11‐months.

At 17 years 6 months of age, her height was at the 50th percentile, weight at the 27th percentile, and head circumference at the 10th percentile. A physical examination was unremarkable except for the following: palpebral fissure lengths 2.2 cm bilaterally (less than −2 *SD*). This is possibly familial as her mother's palpebral fissure lengths measured 2.5 cm bilaterally (also less than −2 *SD*). Interpupillary distance = 5.5 cm (~25th percentile for a 15‐year‐old girl), inner canthal distance = 3.5 cm (at or slightly below +2 *SD*). Length of ears bilaterally: 5.5 cm (−1.2 *SD* for a 16‐year‐old girl). Skeletal: thoracic scoliosis. Extremities: Normal proportions, symmetrical, full ROM at joints. Hyperextensible at 3rd and 4th PIP joints bilaterally. Short left 5th toe. Mild right‐sided calcaneal valgus positioning.

### 1.3 Developmental history

The patient crawled at 10 months of age, walked at 18 months of age, and expressed her first words at approximately 15 months of age. She was enrolled in early developmental therapies as an infant. She was in a special education classroom through the 11th grade. At 15 years of age the patient was neuropsychologically evaluated and determined to be equivalent to a 7 to 8 year old. An IQ test performed at 16 years 6 months of age resulted in a score of 68, consistent with mild intellectual disability. At 17 years 6 months, the patient's math skills were consistent with those of an average 10 year old. There was no history of regression of developmental skills.

## 2 MATERIALS AND METHODS

### 2.1 Ethical compliance

Informed consent was obtained from the family for this article in compliance with institutional and national ethics regulations.

### 2.2 Chromosome analysis

Chromosome analysis was performed on peripheral blood at the 550 band level of resolution, using the GTG‐banding method.

### 2.3 Chromosome microarray analysis

The chromosome microarray was performed, using the Illumina CytoSNP‐850K (GRCh37/hg19 genome assembly) platform on DNA extracted from peripheral blood in the Michigan Medical Genetics Laboratory (http://mmgl.med.umich.edu).

### 2.4 Relative quantitative PCR

The dosage abnormality observed on array was confirmed as previously described by Russell et al. (2014).

### 2.5 Fluorescence in situ hybridization (FISH)

Metaphase FISH analysis was performed at the University of Michigan Clinical Cytogenetics Laboratory on blood samples from the proband's parents, utilizing BAC probe RP11‐89O18 located within the region of interest in 1p34 and a control 1q subtelomeric probe (Abbott Molecular).

## 3 RESULTS

Proband karyotype (20 cells counted, 5 analyzed, and 3 karyotyped) showed 46, XX. Chromosome microarray analysis identified a female chromosome profile with a 2.3 Mb loss of genomic material from the short arm of chromosome 1 at 1p34.3p34.2 ([GRCh37/hg19] Chr1:37973832‐40286005). Relative Quantitative PCR confirmed this deletion (not shown). The deleted region contains a total of 43 RefSeq genes, of which two are OMIM disease genes, 2 are lincRNA genes and 1 is a microRNA gene. The full list of RefSeq genes can be found in Table S1.

To help delineate the origin of this deletion, FISH analysis was performed on the proband's parents. Neither of the proband's parents carried the deletion; therefore, the proband's deletion occurred de novo.

## 4 DISCUSSION

In this report, we describe a female with a de novo 2.3 Mb deletion at 1p34.3p34.2 who has a history of global developmental delay, mild intellectual disability, delayed bone age, bilateral vesicoureteral reflux, vocal cord paralysis, right aberrant subclavian artery, kyphoscoliosis, bilateral metatarsus adductus, and valgus knee deformity.

There are 43 RefSeq genes within the patient's deletion of which two are OMIM disease genes (*SNIP1* and *RSPO1*). The Online Mendelian Inheritance in Man (OMIM) is a continuously updated catalog of human genes and associated genetic disorders. The *SNIP1* gene (OMIM 608241) has been associated with autosomal recessive cognitive impairment, developmental delay, seizures, structural brain abnormalities, and intellectual disability (Puffenberger et al., 2012). These symptoms have only been seen in individuals of Amish descent with a homozygous missense variant. A review of the ClinVar database (August 2017) shows that there has only been one pathogenic variant identified in the *SNIP1* gene. Review of the ExAC database (http://exac.broadinstitute.org/; March 2018) revealed that the *SNIP1* gene is predicted to be severely intolerant to haploinsufficiency with a probability of loss of function intolerance (pLI) of 0.91. The SNIP1 protein interacts with the c‐Myc, NF‐kappa‐B signaling, and TGF‐beta pathways. It has been theorized that haploinsufficiency of the *SNIP1* gene may lead to abnormal brain development and potentially neurodevelopmental delays (Puffenberger et al., 2012; Fujii et al., 2006; Kim et al., 2001, 2000; Dagklis et al., 2016). Tokita et al. (2015), described three individuals whose 1p34.3 deletions also encompass the *SNIP1* gene (probands 1, 2, and 4). All three had a history of motor and speech delays of unknown severity; however, as mentioned previously, their deletions also included the *AGO1* and *AGO3* genes which have been hypothesized to be associated with developmental delays, hypotonia, and poor feeding. We propose that haploinsufficiency of *SNIP1* may be a critical gene underlying our patient's neurodevelopmental delays. However, this does not rule out the contribution of other genes in the deleted region (Table S1) that are also expressed in the brain.

Pathogenic variants in the *RSPO1* gene (OMIM 609595) have been associated with autosomal recessive palmoplantar keratoderma, genitourinary abnormalities, disorder of sex development (46, XX females developing testicular tissue and other extra‐genital findings), and a predisposition to squamous cell skin carcinoma (Radi et al., 2005; Tomaselli et al., 2008; Tallapaka, Venugopal, Dalal, & Aggarwal, 2018). None of the individuals described with a *RSPO1*‐related‐disorder were reported to have developmental delays. According to the ExAC database (March 2018), the *RSPO1* gene has a pLI of 0.00 and thus heterozygous loss of *RSPO1* is less likely to be contributing to our patient's cognitive phenotype.

A review of the Database of Genomic Variants (Build GRCh37, accessed August 2017) revealed that there are no copy number variants in the general population similar in size and genomic endpoints to our patient's deletion. There are many smaller deletions with the largest overlapping deletion being approximately 40 kilobases in size. The DECIPHER database (accessed in March 2018) yields three individuals whose deletions overlap partially with that in our patient (DECIPHER ID's 2750, 4679, 291728). Individuals 4679 and 291728, both have a reported history of developmental delays and hypotonia. Although, in addition to them being haploinsufficient for the *SNIP1* gene, the *AGO1* and *AGO3* are also fully encompassed within their deletions. Individual 2750 has a small deletion (149.87 kb) that contains five RefSeq genes (*FHL3, INPP5B, POU3F1, SF3A3*, and *UTP11*); however, this specific individual seemed to have a more diverse phenotype (abnormal nipple morphology, anteverted nares, delayed speech and language development, downslanted palpebral fissures, epicanthus, episodic vomiting, feeding difficulties in infancy, high palate, hypertelorism, intellectual disability, long phalanx of finger, microcephaly, microtia, pectus carinatum, short stature, small nail) than our patient or any other patient with deletions in this region. Based on what is currently known about these five genes, this deletion seems unlikely to be the cause of all of the individual's symptoms.

To our knowledge, there have not been individuals reported with the 1p34.3p34.2 deletion reported here, that do not also encompass *AGO1, AGO3, GRIK3, SLC2A1,* or *RIMS3*. All five of these genes have been speculated or proven to be associated with neurocognitive delays (Tokita et al., 2015; Takenouchi et al., 2013; Delisi et al., 2002; Vermeer et al., 2007; Lee et al., 2015; Kumar et al., 2009). Clinical similarities between our patient and those described in the literature include the following: intellectual disability, developmental delay, and kyphoscoliosis. Unique characteristics of our patient include delayed bone age, bilateral vesicoureteral reflux, right aberrant subclavian artery, bilateral metatarsus adductus, and valgus knee deformity.

There have been fewer than 10 reported cases with deletions in this region. The fetus presented by Dagklis et al. (2016) had the largest overlapping deletion (overlap by 1.633 Mb) with our patient (Figure S1). Fetal pathological examination found cleft palate, craniofacial malformations (severe posterior micrognathia and microtia), narrow trunk with 11 pairs of thoracic and 1 pair of nuchal sides, abnormal positioning of fingers, talipes varus, knee flexion, dilatation of the fourth ventricle, and malformation of the mitral valve. Other than this fetus and our patient, there have been no other individuals reported with a heart defect who have a deletion of this region.

There are three RefSeq genes deleted in our patient that have not been reported to be deleted in other patients (*KIAA0754, BMP8A, OXCT2P1*). These unique RefSeq genes could be candidates for some of the distinctive features in our patient described in this paper. However, additional patients with deletions in this region need to be ascertained to determine the full clinical impact of heterozygous loss.

## 5 CONCLUSION

Taken together, this patient has a novel 2.3 Mb, de novo, 1p34.3p34.2 deletion that we propose is associated with her symptoms. This adds to the evolving genetic literature that haploinsufficiency of this region and genes other than *AGO1, AGO3*,* GRIK3, SLC2A1,* and *RIMS3* may lead to the neurocognitive delays and other symptoms as identified in our patient. We propose that *SNIP1,* deleted in common with other reported individuals with similar deletions and developmental delays, is a strong candidate gene for the central nervous system pathology. More individuals with comparable deletions are needed to understand the full clinical impact of heterozygous loss of selective genes in this region of chromosome 1.

## References

[mgg3409-bib-0001] Dagklis, T. , Papageorgiou, E. , Siomou, E. , Paspaliaris, V. , Zerva, C. , Chatzis, P. , … Papoulidis, I. (2016). Prenatal diagnosis of 1p34.3 interstitial microdeletion by aCGH in a fetus with jaw bone abnormalities. Molecular Cytogenetics, 9(1), 77. 27713767 10.1186/s13039-016-0288-y PMC5053025

[mgg3409-bib-0002] Delisi, L. E. , Mesen, A. , Rodriguez, C. , Bertheau, A. , LaPrade, B. , Llach, M. , … Sherrington, R. (2002). Genome‐wide scan for linkage to schizophrenia in a Spanish‐origin cohort from Costa Rica. American Journal of Medical Genetics, 114(5), 497–508. 12116183 10.1002/ajmg.10538

[mgg3409-bib-0003] Fujii, M. , Lyakh, L. A. , Bracken, C. P. , Fukuoka, J. , Hayakawa, M. , Tsukiyama, T. , … Roberts, A. B. (2006). SNIP1 is a candidate modifier of the transcriptional activity of cMyc on E box‐dependent target genes. Molecular Cell, 24, 771–783. 17157259 10.1016/j.molcel.2006.11.006

[mgg3409-bib-0004] Kim, R. H. , Flanders, K. C. , Birkey Reffey, S. , Anderson, L. A. , Duckett, C. S. , Perkins, N. D. , & Roberts, A. B. (2001). SNIP1 inhibits NF‐kappa‐B signaling by competing for its binding to the C/H1 domain of CBP/p300 transcriptional co‐activators. Journal of Biological Chemistry, 276, 46297–46304. 11567019 10.1074/jbc.M103819200

[mgg3409-bib-0005] Kim, R. H. , Wang, D. , Tsang, M. , Martin, J. , Huff, C. , de Caestecker, M. P. , … Roberts, A. B. (2000). A novel Smad nuclear interacting protein, SNIP1, suppresses p300‐dependent TGF‐beta signal transduction. Genes and Development, 14, 1605–1616. 10887155 PMC316742

[mgg3409-bib-0006] Kumar, R. A. , Sudi, J. , Babatz, T. D. , Oswald, D. , Yen, M. , Nowak, N. J. , … Dobyns, W. B. (2009). A de novo 1p34.2 microdeletion identifies the synaptic vesicle gene RIMS3 as a novel candidate for autism. Journal of Medical Genetics, 47(2), 81–90. 19546099 10.1136/jmg.2008.065821 PMC2921284

[mgg3409-bib-0007] Lee, M. S. , Kim, Y. J. , Kim, E. J. , & Lee, M. J. (2015). Overlap of autism spectrum disorder and glucose transporter 1 deficiency syndrome associated with a heterozygous deletion at the 1p34.2 region. Journal of the Neurological Sciences, 356(1–2), 212–214. 26117362 10.1016/j.jns.2015.06.041

[mgg3409-bib-0008] Puffenberger, E. G. , Jinks, R. N. , Sougnez, C. , Cibulskis, K. , Willert, R. A. , Achilly, N. P. , … Strauss, K. A. (2012). Genetic mapping and exome sequencing identify variants associated with five novel diseases. Janecke AR, ed. PLoS ONE, 7(1), e28936. 22279524 10.1371/journal.pone.0028936 PMC3260153

[mgg3409-bib-0009] Radi, O. , Parma, P. , Imbeaud, S. , Nasca, M. R. , Uccellatore, F. , Maraschio, P. , … Camerino, G. (2005). XX sex reversal, palmoplantar keratoderma, and predisposition to squamous cell carcinoma: Genetic analysis in one family. American Journal of Medical Genetics, 138A(3), 241–246. 16158431 10.1002/ajmg.a.30935

[mgg3409-bib-0010] Russell, M. W. , Raeker, M. O. , Geisler, S. B. , Thomas, P. E. , Simmons, T. A. , Bernat, J. A. , … Innis, J. W. (2014). Functional analysis of candidate genes in 2q13 deletion syndrome implicates FBLN7 and TMEM87B deficiency in congenital heart defects and FBLN7 in craniofacial malformations. Human Molecular Genetics, 23(16), 4272–4284. 24694933 10.1093/hmg/ddu144

[mgg3409-bib-0011] Takenouchi, T. , Hashida, N. , Torii, C. , Kosaki, R. , Takahashi, T. , & Kosaki, K. (2013). 1p34.3, deletion involving GRIK3: Further clinical implication of GRIK family glutamate receptors in the pathogenesis of developmental delay. American Journal of Medical Genetics, 164(2), 456–460. 10.1111/j.1469-8749.2007.00380.x 24449200

[mgg3409-bib-0012] Tallapaka, K. , Venugopal, V. , Dalal, A. , & Aggarwal, S. (2018). Novel RSPO1 mutation causing 46, XX testicular disorder of sex development with palmoplantar keratoderma: A review of literature and expansion of clinical phenotype. American Journal of Medical Genetics. Part A, 176(4), 1006–1010. 29575617 10.1002/ajmg.a.38646

[mgg3409-bib-0013] Tokita, M. J. , Chow, P. M. , Mirzaa, G. , Dikow, N. , Maas, B. , Isidor, B. , … Prontera, P. (2015). Five children with deletions of 1p34.3 encompassing AGO1 and AGO3. European Journal of Human Genetics, 23(6), 761–765. 10.1002/(ISSN)1096-8628 25271087 PMC4795073

[mgg3409-bib-0014] Tomaselli, S. , Megiorni, F. , Bernardo, C. D. , Felici, A. , Marrocco, G. , Maggiulli, G. , … Grammatico, P. (2008). Syndromic true hermaphroditism due to an R‐spondin1 (RSPO1) homozygous mutation. Human Mutation, 29(2), 220–226. 18085567 10.1002/humu.20665

[mgg3409-bib-0015] Vermeer, S. , Koolen, D. A. , Visser, G. , Brackel, H. J. , van der Burgt, I. , de Leeuw, N. , … de Vries, B. B. (2007). A novel microdeletion in 1(p34.2p34.3), involving the SLC2A1 (GLUT1) gene, and severe delayed development. Developmental Medicine and Child Neurology, 49(5), 380–384. 17489814 10.1111/j.1469-8749.2007.00380.x

