# Supplementary figures and images for: Interstitial microdeletion of the 1p34.3p34.2 region
Source: Mol Genet Genomic Med. 2018 May 3;6(4):673–7. doi: 10.1002/mgg3.409 (PMC6081233; doi:10.1002/mgg3.409)

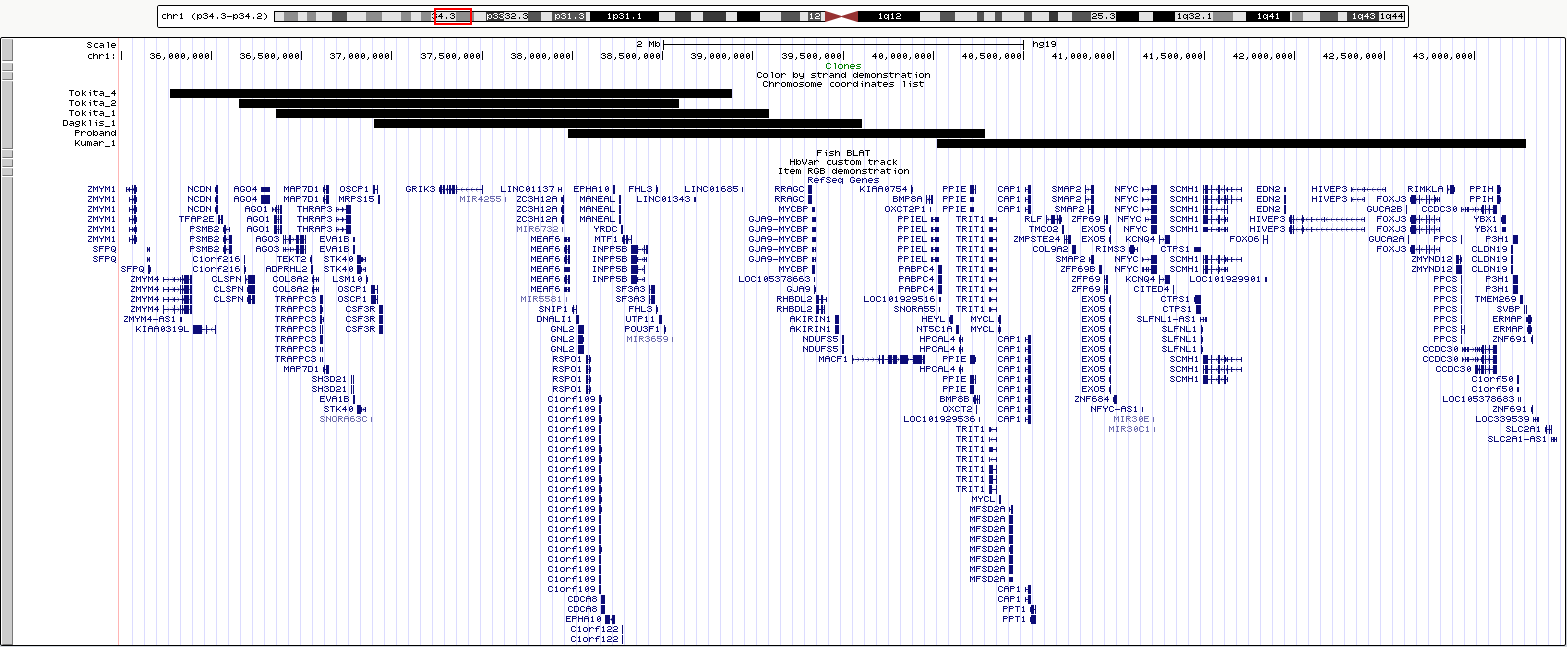

Supplement: Supplementary file 1 [file MGG3-6-673-s001.png]
