# Supplementary material for: Interstitial microdeletion of the 1p34.3p34.2 region
Source: Mol Genet Genomic Med. 2018 May 3;6(4):673–7. doi: 10.1002/mgg3.409 (PMC6081233; doi:10.1002/mgg3.409)
Supplement: Supplementary file 2 [file MGG3-6-673-s002.pdf]

Supplementary Table 1. RefSeq genes in the deleted region

Order	Gene	Cytogenetic Loc	OMIM ID	OMIM Title	Inheritance	Disorders	CNS Expression
1	MEAF6	1p34.3	*611001	MYST/ESA1-ASSOCIATED FACTOR 6; MEAF6			Ubiquitous including brain
2	SNIP1	1p34.3	*608241	SMAD NUCLEAR INTERACTING PROTEIN 1; SNIP1			Ubiquitous including brain
			#614501	PSYCHOMOTOR RETARDATION, EPILEPSY, AND CRANIOFACIAL DYSMORPHISM;PMRED	AR	Psychomotor retardation, epilepsy, and craniofacial dysmorphism, (3);	-
3	DNALI1	1p34.3	*602135	DYNEIN, AXONEMAL, LIGHT INTERMEDIATE POLYPEPTIDE 1; DNALI1			Ubiquitous including brain
4	GNL2	1p34.3	*609365	GUANINE NUCLEOTIDE-BINDING PROTEIN-LIKE 2; GNL2			Ubiquitous including brain
5	RSPO1	1p34.3	*609595	R-SPONDIN FAMILY, MEMBER 1; RSPO1			Not expressed in brain. Expressed in ~15 other tissues. Highly expressed in endometrium
			#610644	PALMOPLANTAR HYPERKERATOSIS WITH SQUAMOUS CELL CARCINOMA OF SKIN AND46,XX SEX REVERSALPALMOPLANTAR HYPERKERATOSIS AND TRUE HERMAPHRODITISM, INCLUDED	AR	Palmoplantar hyperkeratosis with squamous cell carcinoma of skin and sex reversal, (3); Palmoplantar hyperkeratosis and true hermaphroditism, (3);	

6	C1orf109	1p34.3	*614799	CHROMOSOME 1 OPEN READING FRAME 109; C1ORF109			Ubiquitous including brain
7	CDCA8	1p34.3	*609977	CELL DIVISION CYCLE-ASSOCIATED PROTEIN 8; CDCA8			Low brain expression. Primarily expressed in bone marrow, testes, and lymph nodes.
8	EPHA10	1p34.3	*611123	EPHRIN RECEPTOR EphA10; EPHA10			Low brain expression. Primarily expressed in testis, colon, small intestine, and stomach.
9	MANEAL	1p34.3					High brain expression. Ubiquitous elsewhere, but lower compared to brain
10	YRDC	1p34.3	*612276	YRDC DOMAIN-CONTAINING PROTEIN; YRDC			Ubiquitous including brain
11	C1orf122	1p34.3					Ubiquitous including brain
12	MTF1	1p34.3	*600172	METAL-REGULATORY TRANSCRIPTION FACTOR 1; MTF1			Ubiquitous including brain
13	INPP5B	1p34.3	*147264	INOSITOL POLYPHOSPHATE-5-PHOSPHATASE, 75-KD; INPP5B			Ubiquitous including brain
14	SF3A3	1p34.3	*605596	SPLICING FACTOR 3A, SUBUNIT 3; SF3A3			Ubiquitous including brain
15	FHL3	1p34.3	*602790	FOUR-AND-A-HALF LIM DOMAINS 3; FHL3			Ubiquitous including brain
16	UTP11	1p34.3	*609440	UTP11-LIKE PROTEIN; UTP11L			Ubiquitous including brain
17	POU3F1	1p34.3	*602479	POU DOMAIN,			-

				CLASS 3, TRANSCRIPTION FACTOR 1; POU3F1			
18	MIR3659	1p34.3					-
19	LINC01343	1p34.3					No brain expression. Primary expressed in testis and skin.
20	LINC01685	1p34.3	-	-	-	-	-
21	RRAGC	1p34.3	*608267	RAS-RELATED GTP-BINDING PROTEIN C; RRAGC			Ubiquitous including brain
22	MYCBP	1p34.3	*606535	MYC-BINDING PROTEIN; MYCBP			Ubiquitous with low brain expression
23	GJA9-MYCBP	1p34.3					Ubiquitous including brain
24	LOC105378663	1p34.3					Low brain expression. Primary expressed in testis, colon, skin, and stomach.
25	GJA9	1p34.3	*611923	GAP JUNCTION PROTEIN, ALPHA-9; GJA9			Low brain expression. Primary expressed in testis, skin, stomach and colon.
26	RHBDL2	1p34.3	*608962	RHOMBOID-LIKE 2; RHBDL2			Low brain expression. Primary expressed in colon, skin, stomach, testis, esophagus, stomach and urinary bladder.
27	AKIRIN1	1p34.3	*615164	AKIRIN 1; AKIRIN1			Ubiquitous including brain
28	NDUFS5	1p34.3	*603847	NADH-UBIQUINONE OXIDOREDUCTASE Fe-			Highly expressed in heart. Ubiquitous including brain

				S PROTEIN 5; NDUFS5			
29	MACF1	1p34.3	*608271	MICROTUBULE-ACTIN CROSS-LINKING FACTOR 1; MACF1			Ubiquitous including brain
30	KIAA0754	1p34.3					-
31	BMP8A	1p34.3					Primarily expressed in thyroid and endometrium. Very low expression elsewhere
32	OXCT2P1	1p34.3					-
33	PPIEL	1p34.3					Primarily expressed in thyroid. Ubiquitous expression elsewhere including brain
34	PABPC4	1p34.3	*603407	POLYADENYLATE-BINDING PROTEIN, CYTOPLASMIC, 4; PABPC4			Ubiquitous including brain
35	LOC101929516	1p34.3					Ubiquitous including brain
36	SNORA55	1p34.3					-
37	HEYL	1p34.2	*609034	HAIRY/ENHANCER OF SPLIT-RELATED WITH YRPW MOTIF-LIKE PROTEIN; HEYL			Ubiquitous including brain
38	NT5C1A	1p34.2	*610525	5-PRIME-@NUCLEOTIDASE, CYTOSOLIC, IA; NT5C1A			Primarily expressed in heart, brain, adrenal, esophagus, pancreas, prostate
39	HPCAL4	1p34.2					Primarily expressed in brain with some expression in adrenal
40	PPIE	1p34.2	*602435	PEPTIDYL-PROLYL			Ubiquitous including brain

				ISOMERASE E; PPIE			
41	BMP8B	1p34.2	*602284	BONE MORPHOGENETIC PROTEIN 8B; BMP8B			Primarily expressed in thyroid with ubiquitous low expression including brain
42	OXCT2	1p34.2	*610289	3-@OXOACID CoA TRANSFERASE 2; OXCT2			-
43	LOC101929536	1p34.2					-

1
